# Acute kidney injury in intensive care unit patients: a comparison between the RIFLE and the Acute Kidney Injury Network classifications

**DOI:** 10.1186/cc6997

**Published:** 2008-08-28

**Authors:** José António Lopes, Paulo Fernandes, Sofia Jorge, Sara Gonçalves, António Alvarez, Zélia Costa e Silva, Carlos França, Mateus Martins Prata

**Affiliations:** 1 Department of Nephrology and Renal Transplantation, Hospital de Santa Maria, Av. Prof. Egas Moniz, Lisboa 1649-035, Portugal; 2 Department of Intensive Medicine, Hospital de Santa Maria, Av. Prof. Egas Moniz, Lisboa 1649-035, Portugal

## Abstract

**Introduction:**

Whether discernible advantages in terms of sensitivity and specificity exist with Acute Kidney Injury Network (AKIN) criteria versus Risk, Injury, Failure, Loss of Kidney Function, End-stage Kidney Disease (RIFLE) criteria is currently unknown. We evaluated the incidence of acute kidney injury and compared the ability of the maximum RIFLE and of the maximum AKIN within intensive care unit hospitalization in predicting inhospital mortality of critically ill patients.

**Methods:**

Patients admitted to the Department of Intensive Medicine of our hospital between January 2003 and December 2006 were retrospectively evaluated. Chronic kidney disease patients undergoing dialysis or renal transplant patients were excluded from the analysis.

**Results:**

In total, 662 patients (mean age, 58.6 ± 19.2 years; 392 males) were evaluated. AKIN criteria allowed the identification of more patients as having acute kidney injury (50.4% versus 43.8%, *P *= 0.018) and classified more patients with Stage 1 (risk in RIFLE) (21.1% versus 14.7%, *P *= 0.003), but no differences were observed for Stage 2 (injury in RIFLE) (10.1% versus 11%, *P *= 0.655) and for Stage 3 (failure in RIFLE) (19.2% versus 18.1%, *P *= 0.672). Mortality was significantly higher for acute kidney injury defined by any of the RIFLE criteria (41.3% versus 11%, *P *< 0.0001; odds ratio = 2.78, 95% confidence interval = 1.74 to 4.45, *P *< 0.0001) or of the AKIN criteria (39.8% versus 8.5%, *P *< 0.0001; odds ratio = 3.59, 95% confidence interval = 2.14 to 6.01, *P *< 0.0001). The area under the receiver operator characteristic curve for inhospital mortality was 0.733 for RIFLE criteria (*P *< 0.0001) and was 0.750 for AKIN criteria (*P *< 0.0001). There were no statistical differences in mortality by the acute kidney injury definition/classification criteria (*P *= 0.72).

**Conclusions:**

Although AKIN criteria could improve the sensitivity of the acute kidney injury diagnosis, it does not seem to improve on the ability of the RIFLE criteria in predicting inhospital mortality of critically ill patients.

## Introduction

Multiple definitions have until recently been used for acute kidney injury (AKI), and therefore the wide variation in definitions has made it difficult to compare results across studies and populations [1]. Recently, however, the Acute Dialysis Outcome Initiative group proposed a classification for AKI – the Risk, Injury, Failure, Loss of Kidney Function, and End-stage Kidney Disease (RIFLE) classification – in order to have a uniform standard for diagnosing and classifying AKI [2]. The standard defines three grades of severity – risk (Class R), injury (Class I) and failure (Class F) – and two outcome classes – loss of kidney function and end-stage kidney disease [2].

This classification system includes separate criteria for creatinine and urine output. A patient can fulfill the criteria through changes in serum creatinine or changes in urine output, or both. The criteria that lead to the worst possible classification should be used. Class R is considered if there is an increase of serum creatinine X1.5 or an urinary output < 0.5 ml/kg/hour for 6 hours; Class I is considered if there is an increase of serum creatinine X2 or an urinary output < 0.5 ml/kg/hour for 12 hours; and Class F is considered if there is an increase of serum creatinine X3, or in patients with serum creatinine >4 mg/dl if there is an acute rise in serum creatinine of at least 0.5 mg/dl, or a urinary output < 0.3 ml/kg/hour for 24 hours, or anuria for 12 hours (Table 1).

**Table 1:** Risk, Injury, Failure, Loss of Kidney Function, End-stage Kidney Disease classification [2]

Class	GFR criteria	Urinary output criteria
Risk	Serum creatinine × 1.5 or GFR decrease > 25%	< 0.5 ml/kg/hour × 6 hours
Injury	Serum creatinine × 2 or GFR decrease > 50%	< 0.5 ml/kg/hour × 12 hours
Failure	Serum creatinine × 3, GFR decrease > 75% or serum creatinine ≥4 mg/dl with an acute rise > 0.5 mg/dl	< 0.3 ml/kg/hour × 24 hours, or anuria × 12 hours
Loss	Persistent acute renal failure = complete loss of kidney function > 4 weeks	
End-stage kidney disease	End-stage kidney disease > 3 months	

For conversion of creatinine expressed in conventional units to standard units, multiply by 88.4. Patients are categorized on serum creatinine or urinary output, or both, and the criteria that lead to the worst classification are used. Glomerular filtration rate (GFR) criteria are calculated as an increase of serum creatinine above the baseline serum creatinine level. When the baseline serum creatinine is unknown and there is no past history of chronic kidney disease, serum creatinine is calculated using the Modification of Diet in Renal Disease formula for assessment of kidney function [14], assuming a GFR of 75 ml/min/1.73 m^2^.

Several studies have demonstrated that the RIFLE criteria have clinical relevance for the diagnosis of AKI, classifying the severity of AKI and for monitoring the progression of AKI, as well as having predictive ability for mortality in hospitalized patients in general, and patients in the intensive care unit (ICU) setting in particular [3-12]. Nevertheless, a more recent classification for AKI based on the RIFLE system has been proposed by the Acute Kidney Injury Network (AKIN) [13]. This new staging system (Table 2) differs from the RIFLE classification as follows: it reduces the need for baseline creatinine but does require at least two creatinine values within 48 hours; AKI is defined as an abrupt (within 48 hours) reduction in kidney function, currently defined as an absolute increase in serum creatinine ≥0.3 mg/dl (≥26.4 μmol/l), a percentage increase in serum creatinine ≥50% (1.5-fold from baseline), or a reduction in urine output (documented oliguria < 0.5 ml/kg/hour for > 6 hours); risk maps to Stage 1, but it also considers an increase in serum creatinine ≥0.3 mg/dl (≥26.4 μmol/l); injury and failure map to Stages 2 and 3, respectively; Stage 3 also includes patients who need renal replacement therapy irrespective of the stage they are in at the time of renal replacement therapy; and the two outcome classes loss and end-stage kidney disease have been removed.

**Table 2:** Classification/staging system for acute kidney injury [13] modified from the Risk, Injury, Failure, Loss of Kidney Function, End-stage Kidney Disease criteria [2]

Stage	Serum creatinine criteria	Urine output criteria
1	Increase in serum creatinine ≥0.3 mg/dl (≥26.4 μmol/l) or increase to ≥150% to 200% (1.5-fold to 2-fold) from baseline	< 0.5 ml/kg/hour for > 6 hours
2	Increase in serum creatinine to > 200% to 300% (> 2-fold to 3-fold) from baseline	< 0.5 ml/kg/hour for > 12 hours
3^a^	Increase in serum creatinine to > 300% (> 3-fold) from baseline, or serum creatinine ≥4.0 mg/dl (≥354 μmol/l) with an acute increase of at least 0.5 mg/dl (44 μmol/l)	< 0.3 ml/kg/hour for 24 hours, or anuria for 12 hours

Acute kidney injury is defined as an abrupt (within 48 hours) reduction in kidney function, currently defined as an absolute increase in serum creatinine ≥0.3 mg/dl (≥26.4 μmol/l), a percentage increase in serum creatinine ≥50% (1.5-fold from baseline), or a reduction in urine output (documented oliguria < 0.5 ml/kg/hour for > 6 hours). ^a^Individuals who receive renal replacement therapy are considered to have met the criteria of Stage 3 irrespective of the stage they are in at the time of renal replacement therapy.

These modifications were based on the accumulating evidence that small increases in serum creatinine are associated with adverse outcomes, and on the variability inherent in commencing renal replacement therapy and inherent to resources in different populations and countries. Despite the AKIN criteria possibly having greater sensitivity and specificity, it is currently unknown whether discernible advantages exist with one approach towards definition and classification versus the other.

In the present study, we evaluated the incidence of AKI and compared the ability of the maximum RIFLE and of the maximum AKIN within ICU hospitalization in predicting inhospital mortality of critically ill patients.

## Materials and methods

The present study is retrospective, including all patients admitted to the ICU of the Hospital de Santa Maria (Lisbon, Portugal) between January 2003 and December 2006. Variables such as age, gender, race, body weight, history of cardiovascular disease (angina pectoris, myocardial infarction, cerebrovascular disease, and diabetes mellitus), primary diagnosis, Simplified Acute Physiology Score version II, vasopressor use, need for mechanical ventilation or renal replacement therapy, serum creatinine, urine output and outcome were collected from the ICU database and patient medical charts.

Baseline serum creatinine values were unavailable and were estimated by the Modification of Diet in Renal Disease equation [14], as recommended (assuming a lower limit of the normal baseline glomerular filtration rate of 75 ml/min/1.73 m^2^) and previously applied [2,4,9]. In this ICU, serum creatinine is determined at least once a day and urine output is recorded hourly, for all patients. AKI was defined and classified by means of the RIFLE criteria [2] (Table 1) and the AKIN criteria [13] (Table 2). Patients were categorized on serum creatinine or on urine output, or both, the criteria that led to the worst classification were used, and the maximum AKIN and the maximum RIFLE within ICU hospitalization were reported. At least two serum creatinine values within 48 hours were considered to define AKIN stages. The maximum RIFLE was calculated considering the maximum creatinine with reference to the Modification of Diet in Renal Disease equation-estimated creatinine, and the reference creatinine used for AKIN staging was the lowest creatinine within a 48-hour timeframe.

Sepsis was classified in accordance with the American College of Chest Physicians and the Society of Critical Care Medicine consensus [15]. The Simplified Acute Physiology Score version II was used to evaluate illness severity, and was calculated based on the worst variables recorded during the first 24 hours of ICU admission [16]. Inhospital mortality was considered the outcome measure. Chronic kidney disease patients on dialysis and renal transplant patients were excluded from the analysis. Since this was a retrospective and observational study that did not evaluate a specific therapeutic or prophylactic intervention, institutional ethical approval was not required according to our institution's guidelines.

### Statistical analysis

Continuous variables are expressed as the mean ± standard deviation, and categorical variables are presented as the percentage of the number of cases. Comparisons between RIFLE classes or AKIN stages were performed using analysis of variance and the chi-square test for continuous variables and categorical variables, respectively. Multivariate logistic regression analysis was employed to evaluate the association between RIFLE criteria and AKIN criteria with inhospital mortality. Model fit was assessed by the goodness of-fit test, and discrimination was assessed by the area under the receiver operator characteristic (AuROC) curve.

Data are presented as odds ratios (ORs) with 95% confidence intervals (CIs). A two-tailed *P *value < 0.05 was considered significant. Analysis was performed with the statistical software package SPSS 15.0 for Windows (Produtos e Serviços de Estatísticas, Lisboa, Portugal).

## Results

During the study period 703 patients were admitted to the ICU, but 41 of them were chronic kidney disease patients undergoing dialysis and were excluded from the analysis. None of the patients had received a renal transplant. A total of 662 patients (mean age, 58.6 ± 19.2 years; 392 males; 613 Caucasian; mean Simplified Acute Physiology Score version II, 46.3 ± 18.6) were therefore evaluated. Patient baseline characteristics are summarized in Tables 3 and 4.

**Table 3:** Patient baseline characteristics

Variable	Value
Mean age (years)	58.6 ± 19.2
Male (%)	59.2
Caucasian (%)	92.6
Mean body weight (kg)	74.2 ± 16.1
History of cardiovascular disease^a ^(%)	53.2
Medical admission (%)	76.4
Sepsis^b ^(%)	40.9
Baseline serum creatinine (μmol/l)^c^	96.9 ± 37.2
Simplified Acute Physiology Score version II^d^	46.3 ± 18.6
Vasopressors (%)	40
Need for mechanical ventilation (%)	84.7
Mean length of stay (days)	8.2 ± 6.5

^a^Angina pectoris, myocardial infarction, cerebrovascular disease, and diabetes mellitus. ^b^Defined in accordance with the American College of Chest Physicians and the Society of Critical Care Medicine consensus [15]. ^c^Estimated by the Modification of Diet in Renal Disease equation [14], assuming a lower limit of the normal baseline glomerular filtration rate of 75 ml/min/1.73 m^2^. ^d^Calculated based on the worst variables recorded during the first 24 hours of admission [16].

**Table 4:** Patient baseline characteristics and the Risk, Injury, Failure, Loss of Kidney Function, End-stage Kidney Disease (RIFLE) criteria

Variable	No acute kidney injury (n = 372)	Risk (n = 97)	Injury (n = 73)	Failure (n = 120)	*P *value
Mean age (years)	55 ± 19	64 ± 18	63 ± 15	61 ± 16	< 0.0001
Male (%)	55.6	65.9	63	62.5	0.252
Caucasian (%)	93	91.7	91.8	92.5	1.000
History of cardiovascular disease^a ^(%)	48.4	63.9	61.6	54.2	0.023
Medical admission (%)	71.8	78.4	80.8	86.7	0.008
Sepsis^b ^(%)	26.6	39.2	61.6	74.2	< 0.0001
Baseline serum creatinine (μmol/l)^c^	86 ± 24	100 ± 42	107 ± 26	123 ± 50	< 0.0001
Simplified Acute Physiology Score version II^d^	40 ± 15	48 ± 15	51 ± 18	62 ± 21	< 0.0001
Vasopressors (%)	21.5	48.5	63	76.7	< 0.0001
Need for mechanical ventilation (%)	83.6	85.6	86.3	86.7	1.000
Urine output (l)^e^	2.2 ± 0.9	0.5 ± 0.2	0.9 ± 0.5	1.4 ± 1.1	
Serum creatinine at maximum RIFLE		162 ± 35	235 ± 32	395 ± 54	
Need for renal replacement therapy					
% of patients		2	12.3	56.7	< 0.0001
Mean time (days)	5 ± 2	10 ± 8	9 ± 7	0.599	
Mean length of stay (days)	7 ± 8	8 ± 6	9 ± 10	11 ± 12	0.009
Mortality	11	30.9	32.8	55	< 0.0001
Complete renal function recovery^f^	74.6	73.5	55.6	0.053	

^a^Aangina pectoris, myocardial infarction, cerebrovascular disease, and diabetes mellitus. ^b^Defined in accordance with the American College of Chest Physicians and the Society of Critical Care Medicine consensus [15]. ^c^Estimated by the Modification of Diet in Renal Disease equation [14], assuming a lower limit of the normal baseline glomerular filtration rate of 75 ml/min/1.73 m^2^. ^d^Calculated based on the worst variables recorded during the first 24 hours of admission [16]. ^e^At maximum RIFLE (6-hour urine output for risk, 12-hour urine output for injury, and 24-hour urine output for failure). ^f^If the patient returned to their baseline classification within the RIFLE criteria [2].

### Acute kidney injury stratified by the RIFLE and AKIN criteria

AKI occurred in 43.8% of patients with a maximum RIFLE category: risk in 14.7%, injury in 11% and failure in 18.1% (Table 5). According to AKIN criteria, AKI occurred in 50.4% of patients – 21.1% with Stage 1, 10.1% with Stage 2 and 19.2% with Stage 3 (Table 5). AKIN criteria allowed the identification of more patients as having AKI (*P *= 0.018) and classified more patients with Stage 1 (risk in RIFLE) (*P *= 0.003); however, no statistically significant differences were observed for Stage 2 (injury in RIFLE) (*P *= 0.655) and for Stage 3 (failure in RIFLE) (*P *= 0.672).

**Table 5:** Incidence of acute kidney injury stratified by the Risk, Injury, Failure, Loss of Kidney Function, End-stage Kidney Disease (RIFLE) and the Acute Kidney Injury Network (AKIN) definition/classification schemes

RIFLE classification		AKIN classification	
None	372 (56.2%)	None	328 (49.5%)
Risk	97 (14.7%)	Stage 1	140 (21.1%)
Injury	73 (11%)	Stage 2	67 (10.1%)
Failure	120 (18.1%)	Stage 3	127 (19.2%)
Any category	290 (43.8%)	Any stage	334 (50.4%)

Creatinine criteria led to a maximum RIFLE and a maximum AKIN in 64.1% and 67.4% of patients, respectively, whereas in almost 5% of patients it was the urine output criteria that led to a maximum RIFLE and a maximum AKIN. Creatinine and urine output criteria both led to a maximum RIFLE and a maximum AKIN in 30.3% and 27.8% of patients, respectively (Table 6).

**Table 6:** Patients with acute kidney injury classified by creatinine criteria or urine output criteria, or both criteria

	Creatinine (%)	Urine output (%)	Creatinine + urine output (%)
RIFLE classification			
Risk	85.5	5.2	9.3
Injury	68.5	6.8	24.7
Failure	44.2	5	50.8
Any category	64.1	5.6	30.3
AKIN classification			
Stage 1	87.1	3.6	9.3
Stage 2	73.1	7.5	19.4
Stage 3	42.5	4.7	52.8
Any category	67.4	4.8	27.8

Seventy-nine AKI patients (27.2%), defined by the RIFLE classification based on creatinine and urine output criteria, received renal replacement therapy. The requirement of renal replacement therapy was higher in accordance with severity of AKI, defined by the RIFLE classification based on creatinine and urine output criteria (risk, 2%; injury; 12.3%; failure, 56.7%; *P *< 0.0001; AuROC curve = 0.829), and either on creatinine criteria (risk, 2%; injury, 13%; failure, 56.1%; *P *< 0.0001; AuROC curve = 0.818) or on urine output criteria (risk, 0%; injury, 27.5%; failure, 77.6%; *P *< 0.0001; AuROC curve = 0.787).

### Mortality

The overall mortality was 24.3%, and mortality was significantly higher for AKI patients as compared with non-AKI patients, as follows: AKI defined by any of the RIFLE criteria (41.3% versus 11%, *P *< 0.0001; OR = 2.78, 95% CI = 1.74 to 4.45, *P *< 0.0001) or AKIN criteria (39.8% versus 8.5%, *P *< 0.0001; OR = 3.59, 95% CI = 2.14 to 6.01, *P *< 0.0001) (Tables 7 and 8).

**Table 7:** Mortality according to acute kidney injury stratified by the Risk, Injury, Failure, Loss of Kidney Function, End-stage Kidney Disease (RIFLE) and the Acute Kidney Injury Network (AKIN) definition/classification schemes

RIFLE classification		AKIN classification	
None	11%	None	8.5%
Risk	30.9%	Stage 1	30.7%
Injury	32.8%	Stage 2	32.8%
Failure	55%	Stage 3	53.5%
Any category	41.3%	Any stage	39.8%

**Table 8:** Separate multivariate regression analysis for the Risk, Injury, Failure, Loss of Kidney Function, End-stage Kidney Disease (RIFLE) and the Acute Kidney Injury Network (AKIN) classifications

Criteria	Hospital mortality (odds ratio (95% confidence interval))	*P *value	Area under receiver operator characteristic curve
RIFLE criteria (creatinine + urine output)			
Risk	2.69 (1.49 to 4.88)	0.001	0.733
Injury	2.01 (1.03 to 3.89)	0.038	
Failure	3.59 (2.01 to 6.42)	< 0.0001	
Any category	2.78 (1.74 to 4.45)	< 0.0001	
AKIN criteria (creatinine + urine output)			
Stage 1	3.54 (1.97 to 6.37)	< 0.0001	0.750
Stage 2	2.71 (1.33 to 5.53)	0.006	
Stage 3	4.66 (2.49 to 8.73)	< 0.0001	
Any stage	3.59 (2.14 to 6.01)	< 0.0001	
RIFLE criteria (creatinine)			
Risk	2.63 (1.46 to 4.75)	0.001	0.729
Injury	2.12 (1.1 to 4.08)	0.025	
Failure	3.2 (1.8 to 5.7)	< 0.0001	
Any category	2.68 (1.69 to 4.25)	< 0.0001	
AKIN criteria (creatinine)			
Stage 1	3.18 (1.79 to 5.64)	< 0.0001	0.745
Stage 2	2.74 (1.35 to 5.56)	0.005	
Stage 3	3.93 (2.12 to 7.28)	< 0.0001	
Any stage	3.38 (2.05 to 5.57)	< 0.0001	
RIFLE criteria (urine output)			
Risk	1.3 (0.35 to 4.8)	0.689	0.619
Injury	0.83 (0.31 to 2.21)	0.711	
Failure	3.26 (1.74 to 6.13)	< 0.0001	
Any category	2.06 (1.24 to 3.42)	0.005	
AKIN criteria (urine output)			
Stage 1	1.03 (0.3 to 3.58)	0.953	0.612
Stage 2	1.03 (0.35 to 2.99)	0.953	
Stage 3	2.65 (1.45 to 4.84)	0.001	
Any stage	1.9 (1.16 to 3.14)	0.01	

Defined by both creatinine and urine output criteria or by creatinine criteria or by urine output criteria, including age, gender, race, history of cardiovascular disease, medical admission, sepsis diagnosis, illness severity evaluated by Simplified Acute Physiology Score version II, and need for vasopressors or for mechanical ventilation.

The analysis was repeated using the RIFLE or AKIN classification either based only on creatinine criteria or only on urine output criteria. AKI defined by any of the criteria was associated with mortality (RIFLE creatinine, OR = 2.68, 95% CI = 1.69 to 4.25, *P *< 0.0001; AKIN creatinine, OR = 3.38, 95% CI = 2.05 to 5.57, *P *< 0.0001; RIFLE urine output, OR = 2.06, 95% CI = 1.24 to 3.42, *P *= 0.005; AKIN urine output, OR = 1.9, 95% CI = 1.16 to 3.14, *P *= 0.01). RIFLE classes and AKIN stages based on creatinine criteria predicted mortality – whereas when the maximum RIFLE and the maximum AKIN were based on urine output criteria, only Class F and Stage 3 were independently associated with mortality (Table 8).

**Figure 1:**
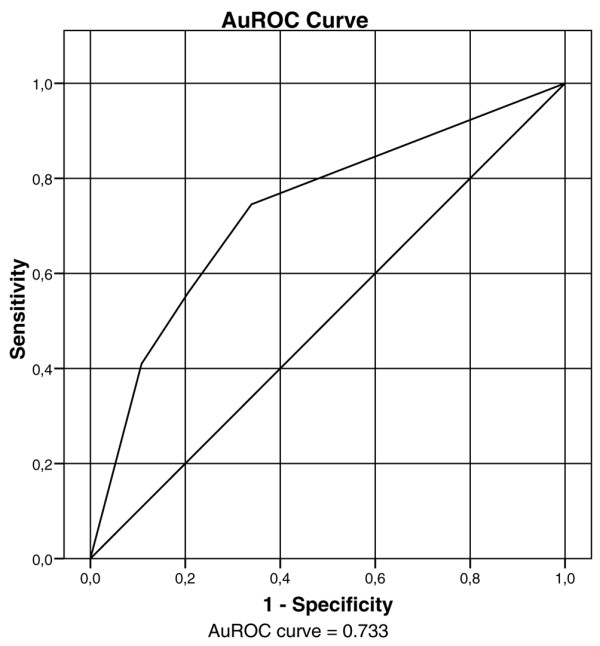
Area under the receiver operator characteristic (AuROC) curve for inhospital mortality for the Risk, Injury, Failure, Loss of Kidney Function, End-stage Kidney Disease criteria (*P *< 0.0001).

**Figure 2:**
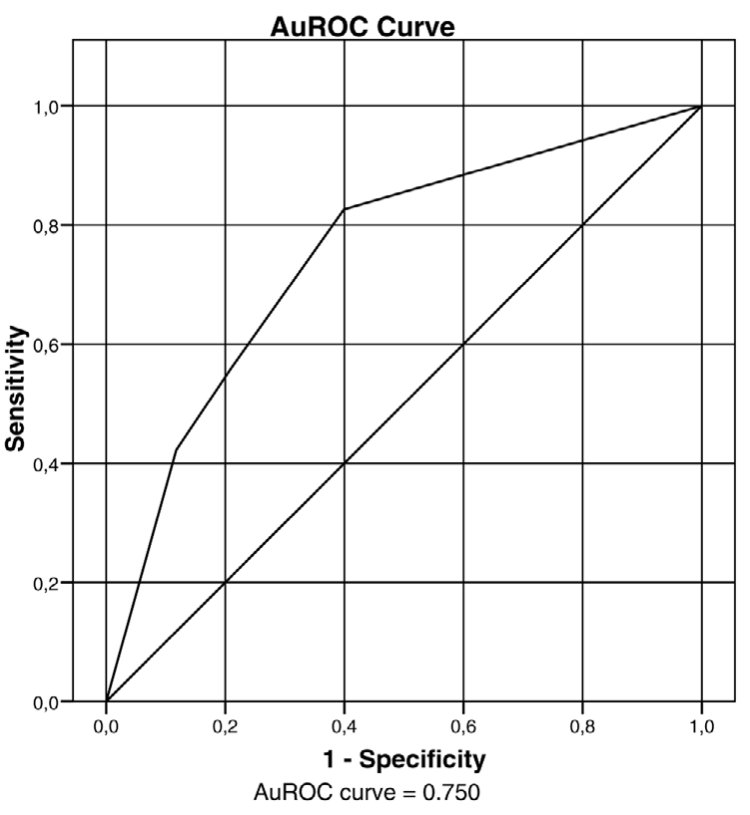
Area under the receiver operator characteristic (AuROC) curve for inhospital mortality for the Acute Kidney Injury Network criteria (*P *< 0.0001).

When considering both creatinine and urine output criteria, the AuROC curve for inhospital mortality was 0.733 for RIFLE criteria (*P *< 0.0001) and 0.750 for AKIN criteria (*P *< 0.0001) (Figures 1 and 2). There were no statistically significant differences in mortality by the AKI definition/classification criteria (*P *= 0.72).

The AuROC curve for inhospital mortality was 0.729 for RIFLE creatinine (*P *< 0.0001) and was 0.745 for AKIN creatinine (*P *< 0.0001), whereas the AuROC curve was 0.619 (*P *< 0.0001) for RIFLE urine output and was 0.612 for AKIN urine output (*P *< 0.0001).

## Discussion

We conducted a single-center study with 662 ICU patients to compare the new recently released definitions/classifications for AKI – the RIFLE system and the AKIN system.

We confirmed that the RIFLE system allows the identification and classification of a significant proportion of ICU patients as having some degree of AKI, and predicts inhospital mortality. These findings have also been reported in a variety of ICU patients [3,5-12].

Nevertheless, a more recent classification for AKI based on the RIFLE system has been proposed by the AKIN workgroup [13]. This new staging system differs from RIFLE as follows: it reduces the need for a baseline creatinine value but does require at least two creatinine values within 48 hours; AKI is defined as an abrupt (within 48 hours) reduction in kidney function, currently defined as an absolute increase in serum creatinine ≥0.3 mg/dl (≥26.4 μmol/l), a percentage increase in serum creatinine ≥50% (1.5-fold from baseline), or a reduction in urine output (documented oliguria < 0.5 ml/kg/hour for > 6 hours); risk maps to Stage 1, but it also considers an increase in serum creatinine ≥0.3 mg/dl (≥26.4 μmol/l); injury and failure map to Stages 2 and 3, respectively; Stage 3 also includes patients who need renal replacement therapy irrespective of the stage they are in at the time of renal replacement therapy; and the two outcome classes loss and end-stage kidney disease have been removed. These modifications were based on the accumulating evidence that small increases in serum creatinine are associated with adverse outcomes, and on the variability inherent in commencing renal replacement therapy and inherent to resources in different populations and countries. Despite the AKIN system possibly having greater sensitivity and specificity, it is currently unknown whether discernible advantages exist with one approach towards definition and classification versus the other.

In the present study, despite AKIN criteria allowing the identification of 6.6% more patients (50.4% versus 43.8%, *P *= 0.018) as having some degree of AKI and increasing the number of patients classified as Stage 1 (risk in RIFLE) (from 14.7% to 21.1%, *P *= 0.003), no statistically significant differences in terms of inhospital mortality were found according to AKI definition/classification criteria. In a recent report Bagshaw and colleagues utilized a large multicenter (120,123 patients, 57 ICUs) clinical database and found no statistically significant differences in terms of incidence of AKI and inhospital mortality by the RIFLE criteria or the AKIN criteria in the first 24 hours after admission [17]. These observations suggest that the proposed modifications for the RIFLE classification, the most widely used definition of acute renal failure in both the critical care and nephrology literature [18], do not improve the ability of this classification in predicting inhospital mortality of ICU patients.

As suggested by a North East Italian multicenter study on AKI, classified by the RIFLE criteria, in 2,164 ICU patients [19], in our analysis the serum creatinine criteria seemed to be a better predictor of mortality than urine output. In fact, a rise in creatinine is an earlier sign of worsening renal function than oliguria. In > 60% of our patients with AKI, the creatinine criteria led to a worse RIFLE class or AKIN stage than urine output.

The current study has some limitations. First, it is a single-center and retrospective study with a relatively small cohort of patients. Second, we did not know the baseline serum creatinine level or the prevalence of chronic kidney disease (except for those undergoing dialysis). Instead, we calculated an estimate of baseline function using the Modification of Diet in Renal Disease equation, as recommended (assuming a lower limit of the normal baseline glomerular filtration rate of 75 ml/min) and previously applied [2,4,9]. Third, despite having hourly records of urine output, we did not have data regarding additional factors that could influence urine output such as diuretic therapy. Overall, we recognize that any biases would influence both the RIFLE criteria and the AKIN criteria, and thus would not significantly influence our conclusions.

Despite these limitations our study has several strengths. First, it is the second study comparing the incidence of AKI, defined by the RIFLE criteria and the AKIN criteria, and the prognostic ability of these classifications in ICU patients. Second, the creatinine criteria and urine output criteria were both used to define and categorize AKI. Finally, we did not limit our analysis to the first 24 hours of ICU admission, contrary to Bagshaw and colleagues [17].

## Conclusion

In summary, our results suggest that although the AKIN criteria could improve the sensitivity of the AKI diagnosis, they do not improve on the ability of the RIFLE criteria in predicting inhospital mortality of critically ill patients. Taking into consideration the extensive validation of the RIFLE criteria in a higher number of patients and heterogeneous groups of patients and cohorts than any other widely accepted and applied definitions and classifications for AKI [20-22], it is time for the utilization of the RIFLE criteria in randomized controlled clinical trials as a surrogate marker of clinically important outcome to establish specific interventions for prevention or attenuation of AKI.

## Key messages

• The RIFLE criteria allowed the identification of 43.8% of ICU patients as having some degree of AKI.

• The AKIN criteria could improve the sensitivity of the AKI diagnosis but do not improve on the ability of the RIFLE criteria in predicting inhospital mortality of ICU patients.

## Abbreviations

AKI: acute kidney injury; AKIN: Acute Kidney Injury Network; AuROC: area under the receiver operator characteristic; CI: confidence interval; ICU: intensive care unit; OR: odds ratio; RIFLE: Risk, Injury, Failure, Loss of Kidney Function, End-stage Kidney Disease.

## Competing interests

The authors declare that they have no competing interests.

## Authors' contributions

JAL, PF, SJ, SG and AA made substantial contributions to the study concept and design, the acquisition of data, and the analysis and interpretation of data. JAL, ZCeS, CF and MMP were involved in drafting the manuscript and revising it critically for important intellectual content. All authors gave final approval of the version to be published.
